# Supplementary material for: Mediating Effects of Specific Types of Coping Styles on the Relationship between Childhood Maltreatment and Depressive Symptoms among Chinese Undergraduates: The Role of Sex
Source: Int J Environ Res Public Health. 2020 Apr 30;17(9):3120. doi: 10.3390/ijerph17093120 (PMC7246430; doi:10.3390/ijerph17093120)
Supplement: Supplementary file 1 [file ijerph-17-03120-s001.pdf]

Supplementary Materials.

Table S1. A Mediating role of different types of coping styles in the association between emotional abuse and depressive symptoms.

Mediator	Model	a	b	Direct Effect	Boot CI		Indirect Effect	Boot CI		Mediation Ratio, %
				c′	LLCI	ULCI	(a × b)	LLCI	ULCI	a × b/(a × b + c′)
**Total**										
Problem solving	1	−0.238 **	−0.959 **	0.767 **	0.699	0.835	0.228	0.198	0.261	13.4
	2	−0.231 **	−0.978 **	0.772 **	0.703	0.840	0.226	0.196	0.259	13.4
Self-blame	1	0.389 **	1.312 **	0.767 **	0.699	0.835	0.511	0.462	0.565	30.0
	2	0.384 **	1.282 **	0.772 **	0.703	0.840	0.493	0.444	0.546	29.2
Help seeking	1	−0.230 **	−0.539 **	0.767 **	0.699	0.835	0.124	0.103	0.147	7.3
	2	−0.230 **	−0.518 **	0.772 **	0.703	0.840	0.119	0.098	0.142	7.1
Fantasy	1	0.253 **	−0.053	0.767 **	0.699	0.835	−0.013	−0.039	0.013	-
	2	0.249 **	−0.007	0.772 **	0.703	0.840	−0.002	−0.027	0.024	-
Problem avoidance	1	0.288 **	0.147 *	0.767 **	0.699	0.835	0.043	0.013	0.072	2.5
	2	0.283 **	0.139 *	0.772 **	0.703	0.840	0.039	0.011	0.068	2.3
Rationalization	1	0.196 **	0.222 **	0.767 **	0.699	0.835	0.044	0.022	0.067	2.6
	2	0.197 **	0.203 **	0.772 **	0.703	0.840	0.040	0.018	0.064	2.4
**Males**										
Problem solving	1	−0.222 **	−1.203 **	0.798 **	0.652	0.943	0.267	0.199	0.350	15.5
	3	−0.220 **	−1.202 **	0.786 **	0.640	0.932	0.264	0.195	0.348	15.6
Self-blame	1	0.370 **	1.457 **	0.798 **	0.652	0.943	0.540	0.433	0.659	31.4
	3	0.360 **	1.418 **	0.786 **	0.640	0.932	0.511	0.408	0.625	30.2
Help seeking	1	−0.227 **	−0.409 **	0.798 **	0.652	0.943	0.093	0.056	0.138	5.4
	3	−0.226 **	−0.425 **	0.786 **	0.640	0.932	0.096	0.060	0.142	5.7

Fantasy	1	0.265 **	−0.089	0.798 **	0.652	0.943	−0.023	−0.084	0.037	-
	3	0.259 **	−0.062	0.786 **	0.640	0.932	−0.016	−0.074	0.043	-
Problem avoidance	1	0.278 **	0.047	0.798 **	0.652	0.943	0.013	−0.050	0.075	-
	3	0.268 **	0.062	0.786 **	0.640	0.932	0.017	−0.042	0.076	-
Rationalization	1	0.182 **	0.180	0.798 **	0.652	0.943	0.033	−0.009	0.078	-
	3	0.173 **	0.181	0.786 **	0.640	0.932	0.031	−0.008	0.074	-
**Females**										
Problem solving	1	−0.241 **	−0.894 **	0.772 **	0.695	0.849	0.215	0.182	0.253	12.6
	3	−0.235 **	−0.901 **	0.765 **	0.688	0.842	0.212	0.179	0.249	12.5
Self-blame	1	0.400 **	1.265 **	0.772 **	0.695	0.849	0.505	0.453	0.563	29.5
	3	0.393 **	1.245 **	0.765 **	0.688	0.842	0.490	0.438	0.546	29.0
Help seeking	1	−0.237 **	−0.555 **	0.772 **	0.695	0.849	0.131	0.106	0.159	7.7
	3	−0.232 **	−0.549 **	0.765 **	0.688	0.842	0.128	0.103	0.154	7.6
Fantasy	1	0.250 **	−0.019	0.772 **	0.695	0.849	−0.005	−0.034	0.023	-
	3	0.246 **	0.010	0.765 **	0.688	0.842	0.003	−0.027	0.030	-
Problem avoidance	1	0.295 **	0.172 *	0.772 **	0.695	0.849	0.051	0.017	0.086	3.0
	3	0.289 **	0.165 *	0.765 **	0.688	0.842	0.048	0.015	0.081	2.8
Rationalization	1	0.205 **	0.221 **	0.772 **	0.695	0.849	0.045	0.020	0.074	2.6
	3	0.206 **	0.220 **	0.765 **	0.688	0.842	0.045	0.019	0.074	2.7

* $p < 0.05$, ** $p < 0.001$. a: Effect of CM on mediators, b: Effect of mediators on depressive symptoms. Model 1: Single factor analysis, Model 2: Adjusted for age, gender, urban/rural, school, only child status, parents' education level, economic status of family, Model 3: Adjusted for age, urban/rural, school, only child status, parents' education level, economic status of family.

Table S2. A Mediating role of different types of coping styles in the association between physical abuse and depressive symptoms.

Mediator	Model	a	b	Direct Effect	Boot CI		Indirect Effect	Boot CI		Mediation Ratio, %
				c′	LLCI	ULCI	(a × b)	LLCI	ULCI	a × b/(a × b + c′)
**Total**										
Problem solving	1	−0.212 **	−1.020 **	0.680 **	0.569	0.790	0.217	0.168	0.269	13.6
	2	−0.213 **	−1.033 **	0.658 **	0.547	0.768	0.220	0.171	0.273	14.4
Self-blame	1	0.343 **	1.415 **	0.680 **	0.569	0.790	0.486	0.408	0.574	30.5
	2	0.326 **	1.387 **	0.658 **	0.547	0.768	0.452	0.376	0.536	29.6
Help seeking	1	−0.208 **	−0.596 **	0.680 **	0.569	0.790	0.124	0.097	0.155	7.8
	2	−0.195 **	−0.584 **	0.658 **	0.547	0.768	0.114	0.088	0.144	7.5
Fantasy	1	0.224 **	−0.009	0.680 **	0.569	0.790	−0.002	−0.025	0.023	-
	2	0.215 **	0.031	0.658 **	0.547	0.768	0.007	−0.016	0.030	-
Problem avoidance	1	0.268 **	0.150 *	0.680 **	0.569	0.790	0.040	0.013	0.071	2.5
	2	0.248 **	0.143 *	0.658 **	0.547	0.768	0.036	0.011	0.064	2.3
Rationalization	1	0.195 **	0.239 **	0.680 **	0.569	0.790	0.047	0.024	0.074	2.9
	2	0.180 **	0.230 **	0.658 **	0.547	0.768	0.041	0.021	0.067	2.7
**Males**										
Problem solving	1	−0.179 **	−1.272 **	0.409 **	0.222	0.596	0.228	0.133	0.342	19.8
	3	−0.175 **	−1.269 **	0.398 **	0.211	0.585	0.222	0.127	0.337	20.0
Self-blame	1	0.255 **	1.569 **	0.409 **	0.222	0.596	0.401	0.269	0.554	34.7
	3	0.241 **	1.527 **	0.398 **	0.211	0.585	0.367	0.244	0.514	33.1
Help seeking	1	−0.197 **	−0.469 **	0.409 **	0.222	0.596	0.092	0.055	0.144	8.0
	3	−0.195 **	−0.486 **	0.398 **	0.211	0.585	0.095	0.057	0.147	8.5
Fantasy	1	0.202 **	−0.027	0.409 **	0.222	0.596	−0.005	−0.054	0.041	-
	3	0.190 **	0.002	0.398 **	0.211	0.585	0.000	−0.045	0.046	-
Problem avoidance	1	0.227 **	0.025	0.409 **	0.222	0.596	0.006	−0.046	0.058	-

	3	0.212 **	0.040	0.398 **	0.211	0.585	0.009	−0.039	0.058	-
Rationalization	1	0.131 **	0.173	0.409 **	0.222	0.596	0.023	−0.006	0.064	-
	3	0.118 **	0.170	0.398 **	0.211	0.585	0.020	−0.005	0.059	-
**Females**										
Problem solving	1	−0.248 **	−0.940 **	0.841 **	0.701	0.980	0.233	0.175	0.296	12.5
	3	−0.240 **	−0.945 **	0.829 **	0.689	0.969	0.227	0.169	0.289	12.5
Self-blame	1	0.396 **	1.365 **	0.841 **	0.701	0.980	0.541	0.447	0.644	29.1
	3	0.384 **	1.342 **	0.829 **	0.689	0.969	0.516	0.425	0.618	28.5
Help seeking	1	−0.202 **	−0.629 **	0.841 **	0.701	0.980	0.127	0.093	0.168	6.8
	3	−0.196 **	−0.621 **	0.829 **	0.689	0.969	0.122	0.089	0.162	6.7
Fantasy	1	0.242 **	0.017	0.841 **	0.701	0.980	0.004	−0.024	0.033	-
	3	0.231 **	0.045	0.829 **	0.689	0.969	0.010	−0.017	0.038	-
Problem avoidance	1	0.288 **	0.190 *	0.841 **	0.701	0.980	0.055	0.022	0.094	2.9
	3	0.274 **	0.181 *	0.829 **	0.689	0.969	0.049	0.018	0.086	2.7
Rationalization	1	0.225 **	0.254 **	0.841 **	0.701	0.980	0.057	0.028	0.092	3.1
	3	0.222 **	0.256 **	0.829 **	0.689	0.969	0.057	0.028	0.091	3.1

* $p < 0.05$, ** $p < 0.001$. a: Effect of CM on mediators, b: Effect of mediators on depressive symptoms. Model 1: Single factor analysis, Model 2: Adjusted for age, gender, urban/rural, school, only child status, parents' education level, economic status of family, Model 3: Adjusted for age, urban/rural, school, only child status, parents' education level, economic status of family.

Table S3. A Mediating role of different types of coping styles in the association between sexual abuse and depressive symptoms.

Mediator	Model	a	b	Direct Effect c′	Boot CI LLCI	Boot CI ULCI	Indirect Effect (a × b)	Boot CI LLCI	Boot CI ULCI	Mediation Ratio, % a × b/(a × b + c′)
**Total**										
Problem solving	1	−0.265 **	−1.023 **	1.108 **	0.934	1.283	0.271	0.199	0.347	12.0
	2	−0.276 **	−1.034 **	1.082 **	0.907	1.256	0.286	0.213	0.364	13.0
Self-blame	1	0.434 **	1.422 **	1.108 **	0.934	1.283	0.618	0.479	0.777	27.4
	2	0.422 **	1.393 **	1.082 **	0.907	1.256	0.588	0.451	0.740	26.7
Help seeking	1	−0.237 **	−0.606 **	1.108 **	0.934	1.283	0.144	0.103	0.192	6.4
	2	−0.220 **	−0.594 **	1.082 **	0.907	1.256	0.131	0.092	0.178	5.9
Fantasy	1	0.292 **	−0.008	1.108 **	0.934	1.283	−0.002	−0.032	0.030	-
	2	0.293 **	0.030	1.082 **	0.907	1.256	0.009	−0.021	0.042	-
Problem avoidance	1	0.349 **	0.149 *	1.108 **	0.934	1.283	0.052	0.017	0.095	2.3
	2	0.336 **	0.140 *	1.082 **	0.907	1.256	0.047	0.014	0.089	2.1
Rationalization	1	0.256 **	0.242 **	1.108 **	0.934	1.283	0.062	0.032	0.102	2.7
	2	0.240 **	0.234 **	1.082 **	0.907	1.256	0.056	0.028	0.095	2.6
**Males**										
Problem solving	1	−0.192 **	−1.257 **	0.829 **	0.550	1.108	0.242	0.119	0.392	14.4
	3	−0.197 **	−1.254 **	0.800 **	0.521	1.079	0.247	0.122	0.394	14,8
Self-blame	1	0.329 **	1.562 **	0.829 **	0.550	1.108	0.514	0.308	0.790	30.6
	3	0.332 **	1.521 **	0.800 **	0.521	1.079	0.505	0.313	0.766	30.3
Help seeking	1	−0.117 **	−0.499 **	0.829 **	0.550	1.108	0.058	0.021	0.112	3.5
	3	−0.125 **	−0.514 **	0.800 **	0.521	1.079	0.064	0.024	0.120	3.9
Fantasy	1	0.267 **	−0.030	0.829 **	0.550	1.108	−0.008	−0.073	0.055	-

	3	0.275 **	−0.002	0.800 **	0.521	1.079	−0.001	−0.067	0.065	-
Problem avoidance	1	0.271 **	0.042	0.829 **	0.550	1.108	0.011	−0.050	0.078	-
	3	0.280 **	0.054	0.800 **	0.521	1.079	0.015	−0.046	0.085	-
Rationalization	1	0.217 **	0.150	0.829 **	0.550	1.108	0.033	−0.014	0.105	-
	3	0.221 **	0.146	0.800 **	0.521	1.079	0.032	−0.015	0.106	-
**Females**										
Problem solving	1	−0.348 **	−0.948 **	1.289 **	1.059	1.519	0.330	0.242	0.434	12.4
	3	−0.341 **	−0.954 **	1.300 **	1.070	1.528	0.325	0.237	0.428	12.3
Self-blame	1	0.507 **	1.382 **	1.289 **	1.059	1.519	0.701	0.516	0.914	26.3
	3	0.499 **	1.358 **	1.300 **	1.070	1.528	0.677	0.501	0.887	25.6
Help seeking	1	−0.308 **	−0.626 **	1.289 **	1.059	1.519	0.193	0.132	0.268	7.2
	3	−0.302 **	−0.618 **	1.300 **	1.070	1.528	0.187	0.128	0.259	7.1
Fantasy	1	0.317 **	0.017	1.289 **	1.059	1.519	0.005	−0.033	0.044	-
	3	0.309 **	0.044	1.300 **	1.070	1.528	0.014	−0.024	0.052	-
Problem avoidance	1	0.396 **	0.179 *	1.289 **	1.059	1.519	0.071	0.026	0.129	2.7
	3	0.389 **	0.168 *	1.300 **	1.070	1.528	0.065	0.021	0.121	2.5
Rationalization	1	0.262 **	0.276 **	1.289 **	1.059	1.519	0.072	0.036	0.120	2.7
	3	0.260 **	0.279 **	1.300 **	1.070	1.528	0.073	0.036	0.120	2.7

* $p < 0.05$, ** $p < 0.001$. a: Effect of CM on mediators, b: Effect of mediators on depressive symptoms. Model 1: Single factor analysis, Model 2: Adjusted for age, gender, urban/rural, school, only child status, parents' education level, economic status of family, Model 3: Adjusted for age, urban/rural, school, only child status, parents' education level, economic status of family.

Table S4. A Mediating role of different types of coping styles in the association between emotional neglect and depressive symptoms.

Mediator	Model	a	b	Direct Effect	Boot CI		Indirect Effect	Boot CI		Mediation Ratio, %
				c′	LLCI	ULCI	(a × b)	LLCI	ULCI	a × b/(a × b + c′)
**Total**										
Problem solving	1	−0.117 **	−0.965 **	0.273 **	0.239	0.306	0.113	0.098	0.129	17.9
	2	−0.119 **	−0.978 **	0.263 **	0.230	0.296	0.116	0.102	0.133	18.9
Self-blame	1	0.115 **	1.402 **	0.273 **	0.239	0.306	0.162	0.141	0.185	25.6
	2	0.112 **	1.379 **	0.263 **	0.230	0.296	0.154	0.133	0.176	25.0
Help seeking	1	−0.107 **	−0.555 **	0.273 **	0.239	0.306	0.059	0.049	0.070	9.4
	2	−0.104 **	−0.546 **	0.263 **	0.230	0.296	0.057	0.047	0.068	9.2
Fantasy	1	0.051 **	0.046	0.273 **	0.239	0.306	0.002	−0.003	0.008	-
	2	0.053 **	0.077	0.263 **	0.230	0.296	0.004	−0.001	0.010	-
Problem avoidance	1	0.090 **	0.129 *	0.273 **	0.239	0.306	0.012	0.003	0.021	1.8
	2	0.089 **	0.120 *	0.263 **	0.230	0.296	0.011	0.002	0.020	1.7
Rationalization	1	0.059 **	0.208 **	0.273 **	0.239	0.306	0.012	0.006	0.020	1.9
	2	0.057 **	0.201 **	0.263 **	0.230	0.296	0.012	0.005	0.019	1.9
**Males**										
Problem solving	1	−0.112 **	−1.205 **	0.223 **	0.157	0.289	0.135	0.103	0.174	23.9
	3	−0.112 **	−1.204 **	0.214 **	0.149	0.280	0.134	0.102	0.173	24.4
Self-blame	1	0.102 **	1.563 **	0.223 **	0.157	0.289	0.160	0.115	0.210	28.2
	3	0.099 **	1.525 **	0.214 **	0.149	0.280	0.151	0.108	0.200	27.4
Help seeking	1	−0.087 **	−0.460 **	0.223 **	0.157	0.289	0.040	0.025	0.059	7.1
	3	−0.087 **	−0.476 **	0.214 **	0.149	0.280	0.041	0.026	0.060	7.5
Fantasy	1	0.072 **	−0.025	0.223 **	0.157	0.289	−0.002	−0.019	0.015	-
	3	0.072 **	0.001	0.214 **	0.149	0.280	0.000	−0.016	0.017	-
Problem avoidance	1	0.089 **	0.020	0.223 **	0.157	0.289	0.002	−0.019	0.022	-

	3	0.088 **	0.033	0.214 **	0.149	0.280	0.003	−0.017	0.023	-
Rationalization	1	0.051 **	0.153	0.223 **	0.157	0.289	0.008	−0.004	0.022	-
	3	0.050 **	0.149	0.214 **	0.149	0.280	0.008	−0.004	0.022	-
**Females**										
Problem solving	1	−0.123 **	−0.891 **	0.288 **	0.250	0.327	0.109	0.092	0.128	16.7
	3	−0.123 **	−0.896 **	0.284 **	0.245	0.322	0.110	0.093	0.129	17.0
Self-blame	1	0.120 **	1.355 **	0.288 **	0.250	0.327	0.162	0.138	0.189	24.7
	3	0.117 **	1.339 **	0.284 **	0.245	0.322	0.156	0.132	0.182	24.2
Help seeking	1	−0.113 **	−0.572 **	0.288 **	0.250	0.327	0.064	0.052	0.078	9.8
	3	−0.111 **	−0.568 **	0.284 **	0.245	0.322	0.063	0.051	0.076	9.7
Fantasy	1	0.043 **	0.090	0.288 **	0.250	0.327	0.004	−0.001	0.010	-
	3	0.045 **	0.108	0.284 **	0.245	0.322	0.005	−0.000	0.011	-
Problem avoidance	1	0.089 **	0.162 *	0.288 **	0.250	0.327	0.014	0.005	0.026	2.2
	3	0.089 **	0.150 *	0.284 **	0.245	0.322	0.013	0.004	0.025	2.1
Rationalization	1	0.059 **	0.224 **	0.288 **	0.250	0.327	0.013	0.006	0.022	2.0
	3	0.060 **	0.229 **	0.284 **	0.245	0.322	0.014	0.006	0.023	2.1

* $p < 0.05$, ** $p < 0.001$. a: Effect of CM on mediators, b: Effect of mediators on depressive symptoms. Model 1: Single factor analysis, Model 2: Adjusted for age, gender, urban/rural, school, only child status, parents' education level, economic status of family, Model 3: Adjusted for age, urban/rural, school, only child status, parents' education level, economic status of family.

Table S5. A Mediating role of different types of coping styles in the association between physical neglect and depressive symptoms.

Mediator	Model	a	b	Direct Effect c′	Boot CI LLCI	Boot CI ULCI	Indirect Effect (a × b)	Boot CI LLCI	Boot CI ULCI	Mediation Ratio,% a × b/(a × b + c′)
**Total**										
Problem solving	1	−0.167 **	−0.982 **	0.488 **	0.431	0.545	0.164	0.140	0.191	15.7
	2	−0.175 **	−0.991 **	0.471 **	0.414	0.529	0.174	0.149	0.201	16.9
Self-blame	1	0.188 **	1.397 **	0.488 **	0.431	0.545	0.263	0.228	0.300	25.0
	2	0.184 **	1.376 **	0.471 **	0.414	0.529	0.253	0.219	0.290	24.6
Help seeking	1	−0.161 **	−0.562 **	0.488 **	0.431	0.545	0.090	0.074	0.108	8.6
	2	−0.154 **	−0.556 **	0.471 **	0.414	0.529	0.086	0.070	0.103	8.3
Fantasy	1	0.078 **	0.059	0.488 **	0.431	0.545	0.005	−0.003	0.014	-
	2	0.085 **	0.085	0.471 **	0.414	0.529	0.007	−0.001	0.017	-
Problem avoidance	1	0.150 **	0.121 *	0.488 **	0.431	0.545	0.018	0.003	0.034	1.7
	2	0.151 **	0.113 *	0.471 **	0.414	0.529	0.017	0.002	0.033	1.6
Rationalization	1	0.102 **	0.206 **	0.488 **	0.431	0.545	0.021	0.009	0.034	2.0
	2	0.099 **	0.202 **	0.471 **	0.414	0.529	0.020	0.009	0.033	2.0
**Males**										
Problem solving	1	−0.148 **	−1.217 **	0.459 **	0.349	0.570	0.180	0.129	0.237	18.1
	3	−0.150 **	−1.215 **	0.440 **	0.328	0.551	0.182	0.133	0.240	18.6
Self-blame	1	0.184 **	1.544 **	0.459 **	0.349	0.570	0.283	0.213	0.367	28.5
	3	0.183 **	1.510 **	0.440 **	0.328	0.551	0.277	0.206	0.359	28.3
Help seeking	1	−0.123 **	−0.462 **	0.459 **	0.349	0.570	0.057	0.034	0.086	5.7
	3	−0.127 **	−0.477 **	0.440 **	0.328	0.551	0.060	0.037	0.090	6.2
Fantasy	1	0.117 **	0.014	0.459 **	0.349	0.570	0.002	−0.025	0.029	-

	3	0.122 **	0.036	0.440 **	0.328	0.551	0.004	−0.023	0.033	-
Problem avoidance	1	0.168 **	−0.011	0.459 **	0.349	0.570	−0.002	−0.041	0.035	-
	3	0.176 **	−0.000	0.440 **	0.328	0.551	0.000	−0.039	0.038	-
Rationalization	1	0.106 **	0.144	0.459 **	0.349	0.570	0.015	−0.009	0.042	-
	3	0.109 **	0.140	0.440 **	0.328	0.551	0.015	−0.010	0.043	-
**Females**										
Problem solving	1	−0.186 **	−0.905 **	0.492 **	0.425	0.559	0.168	0.140	0.200	15.8
	3	−0.186 **	−0.909 **	0.485 **	0.418	0.552	0.169	0.141	0.200	16.1
Self-blame	1	0.187 **	1.357 **	0.492 **	0.425	0.559	0.254	0.213	0.298	23.9
	3	0.184 **	1.342 **	0.485 **	0.418	0.552	0.246	0.205	0.288	23.5
Help seeking	1	−0.170 **	−0.585 **	0.492 **	0.425	0.559	0.100	0.080	0.121	9.4
	3	−0.166 **	−0.581 **	0.485 **	0.418	0.552	0.097	0.078	0.118	9.2
Fantasy	1	0.063 **	0.088	0.492 **	0.425	0.559	0.006	−0.002	0.014	-
	3	0.069 **	0.104	0.485 **	0.418	0.552	0.007	−0.001	0.016	-
Problem avoidance	1	0.138 **	0.164 *	0.492 **	0.425	0.559	0.023	0.008	0.041	2.1
	3	0.140 **	0.152 *	0.485 **	0.418	0.552	0.021	0.006	0.039	2.0
Rationalization	1	0.092 **	0.233 **	0.492 **	0.425	0.559	0.021	0.010	0.036	2.0
	3	0.095 **	0.237 **	0.485 **	0.418	0.552	0.023	0.011	0.038	2.1

* $p < 0.05$, ** $p < 0.001$. a: Effect of CM on mediators, b: Effect of mediators on depressive symptoms. Model 1: Single factor analysis, Model 2: Adjusted for age, gender, urban/rural, school, only child status, parents' education level, economic status of family, Model 3: Adjusted for age, urban/rural, school, only child status, parents' education level, economic status of family.
